# Changes in the ER, PgR, HER2, p53 and Ki-67 biological markers between primary and recurrent breast cancer: discordance rates and prognosis

**DOI:** 10.1186/1477-7819-9-131

**Published:** 2011-10-17

**Authors:** Reiki Nishimura, Tomofumi Osako, Yasuhiro Okumura, Rumiko Tashima, Yasuo Toyozumi, Nobuyuki Arima

**Affiliations:** 1 Department of Breast & Endocrine Surgery, Kumamoto City Hospital, 1-1-60 Kotoh, Kumamoto City, Kumamoto 862-8505, Japan; 2 Clinical Pathology, Kumamoto City Hospital, 1-1-60 Kotoh, Kumamoto City, Kumamoto 862-8505, Japan

**Keywords:** breast cancer, biomarker, Ki-67, discordance

## Abstract

**Background:**

In breast cancer, ER/PgR, HER2, and Ki-67 are important biological markers for predicting prognosis and making effective treatment decisions. In addition, changes in markers due to relapse are also clinically experienced; however, the frequency and clinical significance are still not fully understood. Thus, changes in markers and their correlations with prognosis were investigated.

**Patients and Methods:**

Out of the patients with relapse from 1997 to March 2011, there were 97 consecutive patients from whom the lesion was resected and evaluated by immunostaining. The biopsy sites were chest wall, lymph node, ipsilateral breast tumor recurrence, lungs, bones, ovaries and brain. The markers sought were ER, PgR, HER2, p53 and Ki-67.

**Results:**

The hormone receptor positive rate from the primary tumor to recurrence decreased from 63.9% to 57.7% and from 56.7% to 43.3% for ER and PgR, respectively. Changes in the positive/negative evaluation were seen at the rate of 10.3% and 25.8% for ER and PgR, respectively. The Ki-67 index increased significantly from a mean of 29.1% at primary tumor to 36.3% at relapse. When divided into 2 groups (< 50% and ≥50%), changes were seen in 24.7%. On the other hand, the rates of changes in HER2 and p53 positivity were 14.4% and 12.4%. The changes in subtypes were seen in 25%, however, the lowest rate of change was seen in the triple negative cases. Although there was no notable difference in the rate of change between disease-free interval (DFI) and PgR, Ki-67, p53 and HER2, there was a significant difference in the change rates in the ER. A multivariate analysis revealed that the status of distant metastasis and PgR level at relapse, and Ki-67 levels at primary tumor were all significant factors.

**Conclusion:**

Estrogen receptor and PgR decreased while Ki-67 increased due to relapse; however, the rate of change was high for PgR and Ki-67. Change in the subtypes was seen in 25%. In addition, PgR at relapse and Ki-67 at primary tumor were significant factors for post-relapse prognosis while PgR becoming negative was a poor prognostic factor. These findings are important for making effective treatment decisions.

## Background

Treatment decisions for breast cancer are commonly made based on the information derived from the immunohistochemistry (IHC) of biological markers. For example, the application of chemotherapy is commonly used for patients with high Ki-67 values, endocrine therapy for ER positive, and anti-HER2 (trastuzumab) therapy for HER2 positive have recently been recommended [1]. Post-operative adjuvant treatment decisions are commonly based on the IHC data at surgery. Moreover, treatment decisions for recurrent cases are generally based on ER and HER2 status at primary tumor, disease-free interval (DFI), recurrence site, and the performance status [2]. Recently, surgical resection of recurrent lesions followed by pathological confirmation of relapse and the investigation of the biological markers is also being proposed for effective treatment decisions.

This biological change is due to the progression of cancer and metastasis. The authors in most studies [3-5] have stated that hormone receptors (HRs) change readily while there are few changes in HER2. Furthermore, there are also reports of changes in treatment in conjunction with this change [6]. However, the mechanism of this change is still relatively unknown and there are only a few reports outlining the importance of the Ki-67 index and p53 overexpression. This study attempts to contribute to this endeavor by measuring the biological markers in recurrent breast cancer and then compare them to the status at the primary tumor. Furthermore, the involvement of this change in post-relapse prognosis was also examined retrospectively.

## Patients and methods

Out of all the patients with relapse during the period from 1997 to March 2011, there were 97 consecutive patients from whom the lesion was resected either by surgery or biopsy and evaluated by immunostaining. The baseline information of all the patients with recurrent breast cancer enrolled in this study is presented in Table 1. The age of the patients ranged from 31 to 83 years old (median, 53 years). The median value for DFI was 57.4 months, and the DFI for 45 patients (46.4%) exceeded 5 years. The surgical sites were loco-regional recurrences and ipsilateral breast tumor recurrence (IBTR) for the majority of the patients. Exploration for distant metastasis in the lungs (3 patients), brain (3 patients), bone (1 patient), and ovaries (3 patients) was also performed. However, distant metastasis at surgery for recurrence was explored in 33 (34.0%) patients. The biological markers were as follows: ER positive rate was 63.9%, PgR positive rate was 56.7%, and HER2 and p53 overexpression was 22.7%. On the other hand, the median value of the Ki-67 index was 22.7%, which was slightly higher than the median value of all the primary cases [7]. To explore the biological markers in this study, an informed consent was obtained from all the patients at both the initial surgery and at the relapse biopsy. Moreover, no new primary cases were included in this study.

**Table 1:** Characteristics of all patients with recurrent breast cancer in this study

Characteristics	Number	Percent
Median age at diagnosis (years), range	53 (31 - 83)	
Total	97	
Tumor size at the primary tumor		
≤ 2.0 cm	51	52.6
2.0 cm <	46	47.4
No. of positive nodes at the primary tumor		
0	46	47.4
1-3	27	27.8
4≤	21	21.6
Unknown	3	3.1
Nuclear grade at the primary tumor		
1	16	16.5
2	50	51.5
3	19	19.6
Unknown	12	2.1
Lymphatic invasion at the primary tumor		
0	27	27.8
1+	51	52.6
2+	17	17.5
Unknown	2	2.1
ER at the primary tumor		
Positive	62	63.9
Negative	35	36.1
PgR at the primary tumor		
Positive	55	56.7
Negative	42	43.3
HER2 at the primary tumor		
Positive	22	22.7
Negative	75	77.3
p53 at the primary tumor		
Positive	22	22.7
Negative	75	77.3
Ki-67 index at the primary tumor		
Positive	12	12.4
Negative	85	87.6
DFI at recurrence		
< 2 years	19	19.6
2-5 years	33	34.0
5 years ≤	45	46.4
Sites of biopsied recurrence		
Chest wall	39	40.2
In-breast	34	35.1
Regional lymph node	11	11.3
Lung	3	3.1
Bone	1	1.0
Brain	3	3.1
Ovary	3	3.1
Distant skin	3	3.1
Distant metastasis at biopsy		
With	33	34.0
Without	64	66.0
		
Median Ki-67 value (%) at the primary tumor, range	27.0 (1-91)	
Median DFI (months), range	57.4 (1-281)	

DFI: disease-free interval, ER: estrogen receptor, PgR: progesterone receptor

The post-surgery follow up and examination was performed every 3 months until 3 years post-surgery, every 3-6 months until 3-5 years post-surgery, and every 6-12 months post-surgery from 5 years and onwards. In some cases, the follow-up was continued even after 10 years post-surgery. Mammography, chest X-ray, CT scan or abdominal ultrasound (US), and tumor marker tests are performed at least once a year.

Postoperative adjuvant treatment is being performed in accordance with the treatment recommendations of the St. Gallen International Conference [1]. Endocrine therapy is performed for ER/PgR positive cases and chemotherapy for ER negative cases with or without lymph node metastasis. In addition, trastuzumab treatment has been performed for HER2 positive cases since 2008. For the patients in this study, trastuzumab was not used as an adjuvant therapy. However, it was used for patients with recurrence from 2001. Due to the nature of different treatments that require different lengths of time for effective results, it was difficult to identify the correlation between treatments and the changes in the biological markers. Moreover, recurrence after treatment varies and therefore it is difficult to summarize these treatment patterns.

### Histopathological Examination

The items investigated were the presence or absence of distant metastasis at biopsy or surgery for recurrent lesions, ER/PgR status, proliferation (Ki-67), HER2, and p53 overexpression. Immunostaining of ER, PgR, p53, Ki-67 and HER2 was done as previously described [8].

Biological markers were examined at primary tumor and biopsy or operation for recurrent lesions. The variables of interest were ER, PgR, HER2, Ki-67 labeling index, and p53 overexpression. Hormone receptor positivity was defined by positive staining for estrogen- and/or progesterone-positive receptors in at least 10% of the tumor cell nuclei. HER2 expression was initially evaluated using the Hercep Test (Dako, Glostrup, Denmark). HER2 positivity was indicated by 3+ staining intensity. HER2 equivocal (2+ staining) was tested using fluorescence in situ hybridization with a threshold ratio of > 2.0 for positive HER2:CEP17. Proliferation activity was assessed by immunostaining with the Ki-67 antibody (Dako). The proportion of proliferating cells was determined by counting at least 500 tumor cells. The median value of Ki-67 index was 20% in all primary breast cancer, and the median value of TN tumors was 50% [7]. Moreover, higher values (≥50%) correlated with early recurrence and unfavorable prognosis. Therefore, in this study, the cases were divided into 2 groups; < 50% and 50%≤. Expression of p53 was also evaluated by immunostaining with the mouse monoclonal anti-p53 antibody (clone DO7; Dako). The staining pattern of the p53 protein was divided into three groups, 2+ (homogenous and diffuse staining), 1+ (heterogeneous or focal staining in > 5% of the tumor cells), and negative (focal staining in < 5% of the tumor cells). In this study, p53 overexpression was indicated by 2+ staining. The subtypes were classified as follows: HR positive and HER2 negative tumors were classified as luminal A type; HR positive and HER2 positive tumors (HER2 IHC: 3+ or 2+ and FISH amplification ratio > 2.0) as luminal B type; HR negative and HER2 positive tumors as HER2 disease; and HR negative and HER2 negative tumors as the TN type.

### Statistical Analysis

Statistical comparisons between groups were performed using the Chi-square and Fisher's exact tests. The Wilcoxon's (non-parametric) test was used to compare the mean values for the Ki-67 index. The Kaplan-Meier test was used to calculate prognosis (cumulative overall survival (OS)). Cox's proportional hazard model was used to perform univariate and multivariate analyses of the factors related to OS after recurrence. A two sided p value of < 0.05 was considered to be statistically significant. The median observation period after recurrence was 41.9 months.

## Results

### 1. Changes in positive rate due to relapse of biological markers

Changes due to relapse of a positive rate for each marker are shown in Table 2. There was a decrease in the following hormone receptors; ER from 63.9% to 57.7%, PgR from 56.7% to 43.3%, and there was only a marginal difference for PgR. Moreover, the values for p53 and HER2 changed from 22.7% to 24.7% and from 22.7% to 30.9%, respectively. Regarding Ki-67 index, the positive rate significantly increased from 12.4% to 24.7%. Furthermore, the mean value of 29.1% at primary tumor increased notably to 36.3% at relapse.

**Table 2:** Changes in biological marker status (positive rates) between primary breast tumors and matched recurrent lesions

Biological Markers	Primary	Recurrence	p value
	**Positive rate (%)**		

Estrogen Receptor	62 (63.9)	56 (57.7)	0.46

Progesterone Receptor	55 (56.7)	42 (43.3)	0.08

p53	22 (22.7)	24 (24.7)	0.87

HER2	22 (22.7)	30 (30.9)	0.26

Ki-67 Index	12 (12.4)	24 (24.7)	0.012

(Ki-67: mean ± S.D.(%)	29.1 ± 18.5%	36.3 ± 21.0%	< 0.0001 )

### 2. Changes by category due to relapse of the biological markers

Changes between categories of each marker are shown in Table 3. Changing from positive to negative was common for ER and PgR, with a significant change in PgR in 24.8% of the patients. For p53 and HER2, changing to positive was common, but there was no notable difference. On the other hand, the Ki-67 index had a significantly higher rate of change from negative to positive (22.6%).

**Table 3:** Changes in biological marker status (category) between primary breast tumors and matched recurrent lesions

Marker	Decrease	Increase	% Change	p value	p value
	**primary → recurrence**			* PgR	** Ki-67

ER	+ → - : 8	- → + : 2	10/97 (10.3)	0.009	0.03

PgR	+ → - : 19	- → + : 6	25/97 (25.8)*		0.74

p53	+ → - : 5	- → + : 7	12/97 (12.4)	0.028	0.09

HER2	+ → - : 3	- → + : 11	14/97 (14.4)	0.073	0.20

Ki-67 Index	+ → - : 5	- → + : 17	22/97 (22.6)**	0.74	

ER: estrogen receptor, PgR: progesterone receptor

### 3. Changes in markers and DFI

Category changes for each marker and the correlation with DFI are shown in Table 4. The rate of change was 4.4% for ER in patients with a DFI of at least 5 years, and a significant difference was seen in patients with a DFI of 5 years or less. However, DFI had no clear correlation with PgR, Ki-67, p53, and HER2.

**Table 4:** Changes in biological marker status (category) between primary breast tumor and matched recurrent lesions according to DFI

Marker/DFI	Decrease	Increase	% Change	p
	
	primary → recurrence			value
ER				

DFI < 2y	+ → - : 2	- → + : 1	3/19 (15.8)	

2y < DFI < 5y	+ → - : 4	- → + : 1	5/33 (15.2)	*p = 0.045

DFI > 5y	+ → - : 2	- → + : 0	2/45(4.4) *	DFI < 5y

PgR				

DFI < 2y	+ → - : 2	- → + : 3	5/19 (26.3)	

2y < DFI < 5y	+ → - : 6	- → + : 1	7/33 (21.2)	NS

DFI > 5y	+ → - : 11	- → + : 2	13/45 (28.9)	(0.74)

Ki-67 Index				

DFI < 2y	+ → - : 0	- → + : 5	5/19 (26.3)	

2y < DFI < 5y	+ → - : 2	- → + : 6	8/33 (24.2)	NS

DFI > 5y	+ → - : 3	- → + : 6	9/45 (20.0)	(0.83)

p53				

DFI < 2y	+ → - : 1	- → + : 0	1/19 (5.3)	

2y < DFI < 5y	+ → - : 3	- → + : 2	5/33 (15.2)	NS

DFI > 5y	+ → - : 1	- → + : 5	6/45 (13.3)	(0.56)

HER2				

DFI < 2y	+ → - : 0	- → + : 1	1/19 (5.3)	

2y < DFI < 5y	+ → - : 1	- → + : 6	7/33 (21.2)	NS

DFI > 5y	+ → - : 2	- → + : 4	6/45 (13.3)	(0.28)

DFI: disease-free interval, ER: estrogen receptor, PgR: progesterone receptor

### 4. Changes in molecular subtypes

Changes in subtypes at primary tumor and relapse are shown in Table 5. Looking at the discordance rates, the values for luminal A and luminal B were high at 28.8% and 33.3%, respectively, while the HER2 and Triple Negative (TN) values were low at 18.7% and 16.7%, respectively.

**Table 5:** Changes in breast cancer subtype between primary breast tumors and matched recurrent lesions

Subtype/primary	No. of cases	Recurrent lesions				Discordance %
			
		Luminal A	Luminal B	HER2	TN	
Luminal A	57	41 (71.2)	8 (14.0)	1 (1.8)	7 (12.3)	28.8
Luminal B	6	2 (33.3)	4 (66.7)			33.3
HER2	16	0	2 (12.5)	13 (81.3)	1 (6.3)	18.7
TN	18	1 (5.6)	0	2 (11.1)	15 (83.3)	16.7

Total	97	44	14	16	23	24.7

TN: Triple Negative

### 5. Investigation of prognosis and predictive factors after recurrence

**Figure 1:**
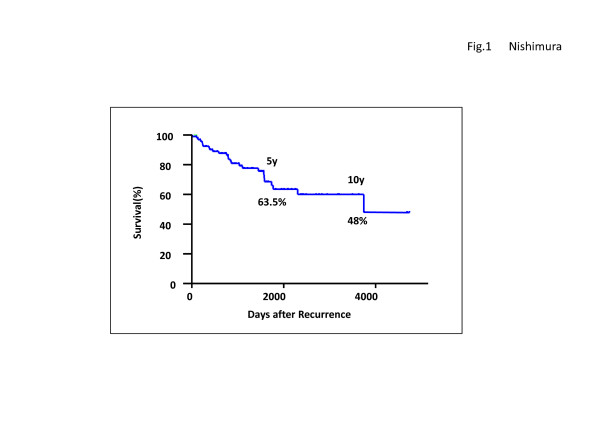
**Overall Survival after Recurrence in the enrolled cases **The rates for the 5- and 10-year overall survival were 63.5% and 48%, respectively, indicating a good prognosis for the study groups.

Cumulative survival rates post-relapse are shown in Figure 1. The rates for the 5- and 10-year OS were 63.5% and 48%, respectively, indicating a good prognosis for the study groups. Univariate and multivariate analyses were performed on the OS-related factors of these patients (Table 6). The univariate analysis revealed that Ki-67 and PgR at primary tumors, and Ki-67, ER, PgR and status of distant metastasis at recurrence were all significant factors. Furthermore, the multivariate analysis revealed that the status of distant metastasis at recurrence, Ki-67 at primary tumor, and PgR at recurrence were all independent factors. All the available data at recurrence was viewed as pertinent information for multivariate analysis.

**Table 6:** Uni- and multivariate analysis of factors for overall survival after recurrence

			Univariate	analysis	Multivariate
			
Variables	Category	primary/recurrence	Relative risk	P value	P value
Primary Tumor					
Tumor size	2.0 cm </≤2.0 cm	primary tumor	0.98	0.93	
Nodal status	0/1-3/4+	primary tumor	1.39	0.15	
Nuclear Grade	1/2/3	primary tumor	1.55	0.22	
Ki-67	50%≤/< 50%	primary tumor	5.67	0.0002	0.0006
ER	+/-	primary tumor	0.49	0.07	0.09
PgR	+/-	primary tumor	0.43	0.037	0.35
p53	+/-	primary tumor	2.18	0.058	0.11
HER2	+/-	primary tumor	0.9	0.82	
Recurrent Tumor					
DFI	5y≤/2y≤/< 2y	at recurrence	0.73	0.22	
Ki-67	50%≤/< 50%	recurrent tumor	2.88	0.009	0.32
ER	+/-	recurrent tumor	0.36	0.013	0.51
PgR	+/-	recurrent tumor	0.25	0.005	0.046
p53	+/-	recurrent tumor	1.78	0.16	
HER2	+/-	recurrent tumor	0.95	0.91	
Distant metastasis	+/-	at recurrence	2.56	0.017	0.043

ER: estrogen receptor; PgR: progesterone receptor; DFI: disease-free interval

*27 cases died of only breast cancer.

## Discussion

In this study, there were many cases of a decreased HR positive rate due to relapse. The rate of change in positive/negative PgR was notable, with a decrease in the rate of positive. On the other hand, the rates of change for p53 and HER2 were low while that of the Ki-67 index category was high at 22.6%, clearly demonstrating a high growth potential due to relapse. At our institution, the IHC evaluation is performed by two pathologists under constant fixed conditions and operating procedures to eliminate the possibility that the difference is due to a difference in evaluation methods. According to recent reports [3-5], changes in ER and PgR due to primary and relapse cancers are common. However, there are only a few changes in HER2. In the investigation of locoregional recurrence (LRR) [9], the discordance rates between primary cancer and LRR was 9% for ER, 22% for PgR and 4% for HER2. In the BRITZ and Destin studies [5], the data of 258 patients (locoregional relapse in 54% and distant metastases in 46%) were compared, and the discordance rates for ER, PgR and HER2 were 13%, 28% and 5%, respectively, showing a notable discordance for PgR. Furthermore, the change in TN cases was reported to be low. On the other hand, in a prospective study [6] on 26 patients with distant metastasis, changes in HR status were observed in 40% (P = 0.003), and for Her2 status, in 8% of the patients. Biopsy results led to a change of treatment in 20% of the patients (P = 0.002). In a retrospective study conducted by the MDACC [10], the changes in ER, PgR, and HER2 were 18.4%, 40.3%, and 13.6%, respectively. In addition, the survival rate for the ER/PgR matching cases was better than the non-matching cases. Furthermore, the discordant rates in a retrospective study [11] on 255 cases of liver metastasis were ER:14.5%, PgR:29.8%, and HER2:13.9%, and the treatment was changed for 18.8% of the cases. The changes in ER and particularly PgR, is notable even in locoregional lesions as well as in distant lesions. On the other hand, the change in HER2 was low. These results were similar to those obtained in the present study. Treatment decisions must take this change into consideration and that is why we select our treatment depending on the condition of the receptors.

Looking at the changes due to treatment based on the Ki-67 index, the number of responders to endocrine therapy as the neoadjuvant therapy decreased, and the prognosis of the patients with decreased levels was good [12]. In addition, the prognosis of patients with decreased levels in post-chemotherapy was reported to be good [13,14]. A decrease in the Ki-67 value was seen in the patients that responded to treatment. Moreover, in the comparison between primary tumors and metastatic lymph nodes, the positive/negative (cutoff point: 20%) matching rate was reported to be 85% [15], and there were only a few changes in many cases where treatment was not involved. Change at relapse in this study was defined as patients who had a relapse after all of the patients received chemotherapy and endocrine therapy, and the relapse lesion did not respond to treatment. Therefore, change in the Ki-67 value can reflect the level of response towards treatment.

Many patients in this study had locoregional lesions which could be biopsied and resected and the post-relapse prognosis was assumed to be good for most of the patients, and in fact, good OS was shown. Investigation of the prognostic factors in such patients using multivariate analysis revealed that the Ki-67 index at surgery and PgR at relapse were significant. PgR turning negative is the first factor found after a relapse, indicating the significance and importance of these evaluations. In addition, looking at the changes in the subtypes of 25% of the patients, the rate of change of the luminal type was notable but the rate for TN was low. However, luminal B in this study was defined as ER+/HER2+. If there is a high Ki-67 value, even in HER2-, which is defined as a criterion for luminal B type cancer, then there will be more changes in many of the patients' subtypes. With regards to the prognosis, the prognosis post-TN relapse was reported to be the worst. Patients with TN breast cancer at LRR experienced a higher risk of subsequent relapse [8]. Post-recurrence survival rates were most favorable in concordant receptor-positive patients for all three receptors. However, patients with discordant receptor status had a similar unfavorable survival rate as patients with concordant TN breast cancer [10]. Although gene expression profiling studies over the past decade have established molecular subtypes, the panel for IHC in a routine pathology setting is a priority because of the current costs of such molecular testing. The IHC is merely a surrogate method of indicating such subtypes. Further research is needed to investigate the relationship between molecular subtypes and changes in biological markers.

## Conclusions

In conclusion, ER and PgR decreased while Ki-67 tended to increase due to relapse; however, the rate of change was high for PgR and Ki-67. In addition, patients with a change in category had low levels for ER/p53/HER2 at approximately 10%, but high levels for PgR and Ki-67 at 25.8% and 22.6%, respectively. On the other hand, changes in ER were significantly fewer in patients with a DFI > 5 years. Subtype changes due to relapse was seen in 25% of the patients. Multivariable analysis revealed that Ki-67 at primary tumor and PgR at relapse are significant factors in post-relapse prognosis. Thus, though changes in biological markers occur due to relapse, pathological confirmation as well as exploration for markers are important. It is particularly important to note that PgR and Ki-67 change readily. A prospective study is necessary to assess the clinical significance of the changes in biological markers.

## Competing interests

The authors declare that they have no competing interests.

## Authors' contributions

RN carried out design of the study, data collection, data analysis, and drafted manuscript. TO, YO, and RT participated in the design of the study and reviewed manuscript. YT and NA carried out the pathological evaluation, and reviewed manuscript. All authors have read and approved the manuscript.
